# Influence of Alkaline Treatment and Fiber Morphology on the Mechanical, Physical, and Thermal Properties of Polypropylene and Polylactic Acid Biocomposites Reinforced with Kenaf, Bagasse, Hemp Fibers and Softwood

**DOI:** 10.3390/polym17070844

**Published:** 2025-03-21

**Authors:** Zeinab Osman, Mohammed Elamin, Elhem Ghorbel, Bertrand Charrier

**Affiliations:** 1 Institute for Engineering Research and Materials Technology, National Center for Research, Khartoum P.O. Box 2404, Sudan; wdalbadawi@yahoo.co.uk; 2 University of Pau and the Adour Region, E2S UPPA, CNRS, Institute of Analytical Sciences and Physico-Chemistry for the Environment and Materials-Xylomat, (IPREM-UMR5254), 40004 Mont de Marsan, France; 3 CY Cergy Paris Université, 5 Mail Gay-Lussac, Neuville-sur-Oise, 95031 Cergy-Pontoise, France; elhem.ghorbel@cyu.fr

**Keywords:** kenaf, bagasse, hemp, PP and PLA, softwood, natural fibers alkaline treatment, thermoplastics biocomposites

## Abstract

This novel study explores a comprehensive approach, combining fiber and matrix structure–property relationships. By integrating alkali treatment, fiber mapping, and intrinsic fiber properties, this work offers a unique perspective on the mechanical, physical, and thermal properties of biodegradable composites of reinforced polypropylene (PP) and plasticized poly (lactic acid) (PLA), with 25 wt% Kenaf (KBF), Bagasse, Hemp fibers and softwood fibers serving as a control. To enhance fiber–matrix interaction, fibers underwent alkaline treatment using 5% sodium hydroxide (NaOH) for one hour. The mechanical properties, including tensile strength, Young’s modulus, and impact strength, were evaluated alongside physical and thermal properties such as fiber mapping, brightness, heat deflection temperature (HDT), melting temperature, melt flow ratio (MFR), and melt flow index (MFI). Scanning electron microscopy (SEM) was used to assess the biocomposites’ morphology. The results showed that fiber reinforcement improved the tensile and impact strength of PP composites, particularly for treated Bagasse (6.6% and 22%) and Hemp (7% and 44.7%), while Kenaf exhibited minimal change, indicating its inherently high strength. A slight increase in tensile strength and Young’s modulus was observed in all PLA-based composites. The addition of 25% fiber enhanced the thermal properties of both treated and untreated fiber-reinforced composites. Among PP composites, those reinforced with treated fibers exhibited the highest HDT, with Kenaf achieving the best performance (124 °C), followed by Bagasse (93 °C). The HDT values for untreated fibers were 119 °C for KBF, 100 °C for softwood, 86 °C for Bagasse, and 79 °C for Hemp. PLA composites showed a slight increase in HDT with fiber reinforcement. Differential Scanning Calorimetry (DSC) revealed a slight decrease in melting temperature for PP composites and a slight increase for PLA composites. Fiber mapping analysis indicated that Kenaf had the highest aspect ratio, contributing to superior mechanical performance, while Hemp had the lowest aspect ratio and exhibited weaker mechanical properties. Overall, Kenaf and Bagasse fibers demonstrated superior mechanical and thermal properties, comparable to those of softwood fibers, whereas Hemp exhibited moderate performance. The variations in composites behavior were attributed to differences in fiber mapping, alkaline treatment, and the intrinsic properties of both the polymer matrices and the reinforcing fibers. These findings highlight the potential of treated natural fibers, particularly Kenaf and Bagasse, in enhancing the mechanical and thermal properties of biodegradable composites, reinforcing their suitability for sustainable material applications.

## 1. Introduction

Natural fiber-reinforced polymer composites (NFRPCs) have attracted significant interest among researchers in recent years due to the need to develop an environmentally friendly material, and partly replacing currently used glass for composite reinforcement [1]. Natural fibers have advantages over synthetic or manmade fibers such as glass and carbon due to their low cost, low density, acceptable specific strength properties, ease of separation, carbon dioxide sequestration, and biodegradability [2].

Significant research efforts have been made and to develop natural fiber-reinforced composites, which are also described as biocomposites in which either of the constituents should be derived from renewable resources [3]. These composites have better mechanical properties like a better strength to weight ratio, high modulus, high specific strength, partial biodegradability characteristics, better chemical resistance property, better insulation performance, and low cost [4,5]. Due to this extraordinary set of properties, NFRPCs have widespread engineering and emerging applications [6,7], and therefore they have become a continuously developing research field.

NFRPCs continue to attract significant attention, especially when they compared with synthetic composites such as carbon fiber-reinforced polymers (CFRPs) and fiber-reinforced thermoplastic (FRTP) composites, which both are widely used for their high strength, stiffness, and resistance to corrosion and fatigue. However, their bonding performance and durability face challenges due to material limitations, joint geometry variations, and harsh environmental effects. These factors can significantly impact the effectiveness of CFRP strengthening systems. It has been found that CFRPs with better mechanical properties demonstrate a superior bond performance when the same adhesive is used [8].

Polypropylene (PP) composites made from carbon and glass fibers offer numerous advantages such as high strength and corrosion resistance, and these composites are utilized in numerous industries, including the automotive, aerospace, and construction industries [9]. However, they also have some limitations, such as their relatively low resistance to UV radiation and high temperatures, which can lead to degradation and reduced mechanical performance over time. Additionally, PP composites have limited compatibility with polar fillers and reinforcements due to the non-polar nature of PP, often requiring surface treatments or compatibilizers to improve bonding. Their low impact strength at sub-zero temperatures and moderate wear resistance may also limit their use in extreme environments [10].

Furthermore, recycling PP composites remains challenging, particularly when multiple reinforcements or additives are used, complicating material recovery and sustainability efforts [9]. Moreover, these synthetic reinforcements, such as glass fibers, pose health risks and can cause wear on manufacturing equipment.

With the growing demand for innovative, sustainable, and recyclable strengthening materials for long-lasting structures, fiber-reinforced thermoplastic FRTP composites offer superior fracture toughness and durability. Their recyclability, enabled by reversible physical processes such as heating, melting, and cooling, has minimal effect on their mechanical properties and microstructure. These advantages make thermoplastic composites particularly suitable for strengthening applications, especially in severely adverse environments [11].

Among various thermoplastic polymers, Polypropylene (PP) and polylactic acid (PLA) have emerged as an interesting matrix with moderate physical and mechanical properties. Polypropylene is one of the lightest of all thermoplastics, with a density of 0.9 g/cm^3^. In addition, its high melting temperature (160–170 °C) makes it heat-resistant and provides it with an ability to withstand various molding techniques and environments. Furthermore, it has excellent mechanical properties such as fatigue and resistance and strong chemical resistance toward most organic solvents. PP’s recyclability became a critical attribute in its widespread use. Recycled PP maintains its properties, making it a viable option for ecologically responsible applications [12].

These properties make it well suited for high-performance fiber-reinforced thermoplastic (FRTP) composites as strengthening materials [11]. Hence, it is an obvious choice as the matrix material in the preparation of natural fiber-reinforced composites [13,14].

Among bio-based polymers, PLA has demonstrated significant potential and commercial value across various industries. It is a compostable, transparent synthetic polymer derived from renewable resources like rice, corn starch, potatoes, sugar beets, or sugarcane. PLA is produced either through ring-opening polymerization of lactide or condensation polymerization of lactic acid [15,16]. PLA has attractive properties such as biodegradability, biocompatibility, and mechanical strength, and its recyclability is an advantage which reduces disposable waste and therefore makes it more economical [17].

In contrast, the use of PP and PLA in NFRPCs provide numerous advantages over traditional mineral reinforcements, including lower production costs, reduced energy consumption, lighter weight, environmental sustainability, and a positive contribution to global sustainable development goals [18].

However, researchers working on NFRPCs are facing some challenges, such as the poor mechanical properties associated with the known hydrophilic nature of the natural fibers. Furthermore, the heterogeneous characteristics of natural fiber lead to a wide variation in fiber quality and relatively poorer mechanical properties, leading to incompatibility and aggregation tendency in hydrophobic polymer matrix and low thermal stability. Thus, selection of a suitable natural fiber is the one of the most important factors that influence the performance of the NFRPC [19].

Currently, kenaf and hemp are widely used in the production of NFRPCs. Both fibers are annual plants and have long bast fibers representing 40% and 70% of their stem’s weight, respectively [20,21]. On the other hand, softwood is typically preferred for thermoplastic composites due to its larger aspect ratio [22]. Sugarcane is grown worldwide as an agricultural crop, whose residue, after the extraction of juice, is referred to as bagasse. It is widely available as an agro-residue, and biocomposites derived from such renewable resources offer potential for scale-up and value addition [23].

Much work has been conducted based on virgin thermoplastic and natural fiber composites, successfully supporting their potential across a wide range of applications in several industrial sectors [24]. Although these four types of natural fibers have the edge in composite manufacturing due to their robustness, many factors can prevent them from displaying their full potential due to resin-reinforcement incompatibility and the presence of surface impurities. Chemical treatment is a well-known method employed to clean the surfaces of fibers and remove unwanted components, such as waxes, pectin, hemicellulose, and lignin, which help to improve interfacial bonding with the commonly used industrial resins [25]. A lot of work has been performed by many researchers with regard to fiber chemical treatment such as alkali, silane, acetylation, alkaline hydrogen peroxide, benzoylation, acrylation, and acrylonitrile graftin and isocyanate to improve the mechanical properties of NFRPCs [26]. Out of these methods, it has been observed that one of the simplest, most economical, and effective forms of treatments with least environmental impact is alkali treatment, particularly mercerization using NaOH.

Much work has been conducted involving alkali-treating kenaf with 6% of NaOH solution for 24 h [27] and hemp [28] for 48 h at 20 °C using different percentages for use in composites. The authors observed that better fiber–matrix adhesion led to an increase in interfacial energy and in the percentage of the OH group which may result in more reaction sites for the fiber–matrix adhesion and thus enhance the thermal and mechanical properties of the composites. High NaOH concentration was reported to cause excess delignification of fibers, resulting in the weakening of the fiber; reduce the degree of polymerization; and minimize lignin content and hemicellulose within the fiber [29]. For example, 1% of NaOH-treated bagasse fiber-reinforced composites showed significant improvement in flexural, tensile, and impact strengths compared to 3 and 5% NaOH-treated fibers [30].

It can be concluded that when seeking to improve the mechanical properties of fiber-reinforced thermoplastic composites, proper fiber treatment is an effective strategy. However, chemical treatments can be advantageous, and easy-to-use, cost-effective reagents such as sodium hydroxide have been used without severe environmental impact and with good processability; such treatments could easily be scaled up and made commercially viable [31]. Hence, treating the fibers with 5% NaOH for one hour was tested in a previous research work, and the authors concluded that mild NaOH concentrations between 0 and 10 wt % improve fiber crystallinity by removing amorphous materials and increasing cellulose content. The removal of lignin also enhances fiber morphology, fostering individualization and higher aspect ratios, which boost mechanical properties.

However, concentrations exceeding 10 wt % may cause excessive delignification, compromising the fiber cell wall and diminishing the fibers’ properties. Moreover, composites made with fibers treated at 10 wt % NaOH exhibit slightly higher flexural modulus values compared to those treated at lower NaOH concentrations of 5 and 7.5 wt %. These findings were supported by a recent study on the glass fiber-reinforced polypropylene (GFPP), which revealed that prolonged immersion in alkaline solutions resulted in a significant reduction in tensile strength [32]. Mild NaOH treatments produce less waste, supporting circular economy objectives and green chemistry principles [33].

In this paper, the aim was to study the effect of fiber morphology, intrinsic properties, and alkali treatment with NaOH on the mechanical, physical and thermal properties of the injection-molded PP and PLA composites reinforced with kenaf bast fibers, Bagasse, and hemp. This novel combination provides deeper insights into the behavior and performance of natural fiber-reinforced biocomposites, which have not been extensively explored in previous studies. Furthermore, their properties were compared to the softwood. A comparison was also made between the intrinsic properties of the PP, PLA, and the fibers used. Therefore, our major goal was to develop sustainable and partially/completely eco-friendly thermoplastic materials based on PP and PLA in order to obtain desired mechanical and thermal properties. The end-product made of this composite’s material may undergo both energetic and material recycling and has a lower carbon footprint. The main limitation of this study is that only a single alkaline treatment condition (5% NaOH for one hour) was used, without exploring variable concentrations or treatment durations. Additionally, fiber mapping was not conducted after alkali treatment, which could have provided deeper insights into structural changes. However, these aspects will be thoroughly investigated in future research to enhance our understanding of the treatment’s effects on fiber properties and composite performance.

## 2. Materials and Methods

### 2.1. Materials

#### 2.1.1. Lignocellulosic Materials

The kenaf was planted at the demonstration farm of Khartoum University, Sudan. Kenaf bast fibers (KBFs) were water retted for 10 days in open tanks and then dried at room temperature according to a method reported by Bledzki, A. et al., 2015 [17]. Bagasse was collected from White Nile Sugar Factory at Algenaid in the White Nile State, Sudan. The bagasse fibers were air dried at room temperature. The hemp fibers used in this study were Etype SKF2, Superkurzfaser, and were purchased from Bafa neu GmbH, Malsch, Germany. The softwood (BK4090) was supplied by Rettenmaier & Söhne GmbH + CO KG; their color was yellow and the particle size ranged from 300 to 500 μm.

##### Chemical and Mechanical Properties of the Lignocellulosic Materials

The chemical composition of the three fibers and their basic mechanical properties are shown in Table 1.

#### 2.1.2. Polymer Matrices

Polypropylene (PP) purchased from Borealis, Vienna, Austria, type BH345MO; CAS;9003-56-9 (an injection molding-grade copolymer) was used as a matrix. This copolymer exhibits good stiffness and high fluidity of MFR = 45 (http://www.matweb.com/tools/unitconverter.aspx?fromID=149&fromValue=45, 23 February 2025) g/10 min (230 °C/2.16 Kg), a melting temperature of 210–260 °C, and a heat deflection temperature of 85 °C. As for PP’s mechanical properties, it has tensile strength of 23.4 MPa and a Young’s modulus of 1290 MPa.

Maleic acid anhydride grafted PP wax (MAH-g-PP, CAS 25722-45-6) from Merck, Lyon, France, of 3 wt %, respectively, was applied to the matrix as a compatibilizer between non-polar matrix and polar lignocellulosic fibers.

Polylactic acid (PLA) of grade 3251D; CAS;9051-89-2 from Natureworks LLC, Neasden, Netherlands, was used in this study. It has a tensile strength of 48.0 MPa, a Young’s modulus of 3630 MPa, flexural strength 83.0 MPa, impact strength of 16 (J/m), and MRF of 35 g/min.

#### 2.1.3. Sodium Hydroxide

NaOH pellets, with a concentration of ≤100%, were purchased from Merck.

### 2.2. Methods

#### 2.2.1. Fibers Preparation

Kenaf fibers were manually cut to the size of 5 mm. Bagasse fibers were reduced using a laboratory hummer mill. They were then screened and particles retained on mesh size 20 (particle size ≈ 1 mm) were further ground with kenaf and hemp using a cutting Mill SM 300. The fibers’ geometry was determined as described in Section 2.2.3.

#### 2.2.2. Fibers Treatment

All fibers were soaked in sodium hydroxide solution with a 5% concentration for 1 h. They were then thoroughly washed with tap water in order to remove any sodium hydroxide that might have stuck to their surfaces (the pH of the last wash was 7). Thereafter, they were dried at 100 °C for overnight prior to their blending with the matrices.

#### 2.2.3. Fiber Shaping

**Figure 1:**
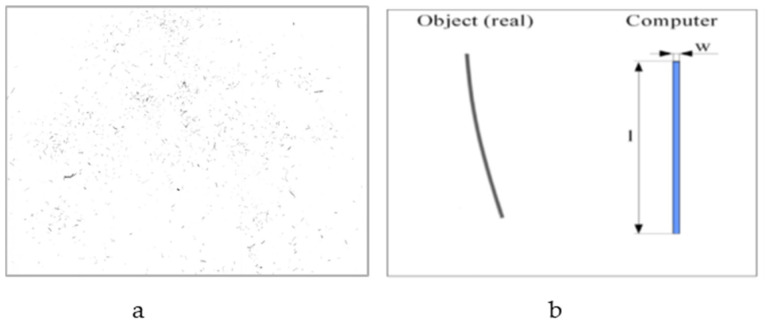
Sample of scanned kenaf SM300 fibers, 1200 dpi (**a**) and rectangle model of fibers (**b**).

The fiber samples were analyzed using a flatbed scanner (Epson Perfection 4990 Photo) at a resolution of 1200 dpi. Each sample was carefully transferred onto the scanning surface using a wooden spatula. To ensure the fibers were evenly dispersed and avoid clumping, a pipette was used to blow air over them, as shown in Figure 1a.

A film holder was used to divide the scanning area into two sections, allowing for an organized layout during scanning. The images captured by the scanner were processed using Fibreshape software (IST AG, St. Gallen, Switzerland). The software analyzed the images (14 images/sample) with a measurement mask specifically designed for this purpose, called “Example Wood Shred 1200 dpi”. The number of fibers used per sample was 26,893.

During the analysis, the software identified and measured all fibers based on predefined algorithms. The minimum measurable fiber thickness was set at 40 µm, which corresponds to the resolution limit of the chosen 1200 dpi setting. The software characterized each fiber as an ideal rectangle, as illustrated in Figure 1b.

Percentile analysis was also included to assess the distribution of fiber dimensions, providing a clearer understanding of the variability within the sample.

#### 2.2.4. Compounding of PP and PLA

**Figure 2:**
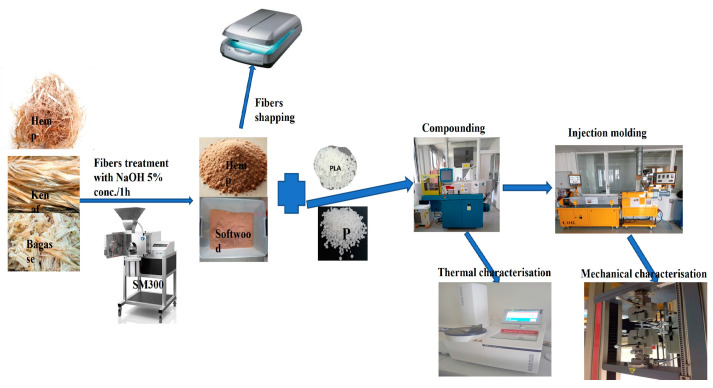
Schematic diagrams of biocomposites preparation.

Figure 2, shows the schematic diagram for the preparation of the biocomposites. Fibers were used with their initial moisture content (kenaf 6.9, bagasse 3.50, softwood (BK4090) 9.46 and hemp 12.1%). PP was processed without drying. The microfibers were mixed with PP and MAH-g-PP granulate in the hopper feeder and were conveyed to the extruder’s feed section (using a counter-rotating tight intermeshing twin-screw extruder from Krauss Maffei Berstorff ZE34 basic L/D = 46 D = 34 mm). Extruded strands were cooled in a water bath and palletized. Materials were compounded at temperatures ranging 190–220 °C RPM.

PLA was compounded with the same fibers at the same moisture content at a temperature ranging between 165 and 180 °C.

The process ensured good fiber distribution and an even fiber–matrix ratio in order to maintain a homogenous density and small deviation of mechanical properties for each manufactured type of composite material. All the samples were conditioned at 23 °C and 50% relative humidity for 16 h prior to performing testing.

#### 2.2.5. Injection Molding

Granules were injection-molded using a machine model KM50-180AX (clamping force 485 kN) and molds in accordance with EN ISO 294-1 [37]. The barrel temperatures were 150–200 °C from feed zone to nozzle. The injection pressure was 550 and 1200 bar at injection speeds of 35 and 40 mm/s for injections with PP and PLA, respectively.

#### 2.2.6. Characterization of Composites

##### Density Measurement

The density of the manufactured composites was measured at room temperature according to EN ISO 1183-1 [38] on a high-accuracy balance Mettler Toledo EL204IC. Samples were immersed in ethanol with its density determined before each series of measurements on the very same balance. Each weighing process was repeated three times and averaged.

##### Brightness Measurement

The injection molded samples were scanned using a standard spectrophotometer-spectro-guide 45/0 gloss S BYK-Gardner. The measuring device was placed on the even sample surface and the L*, a* and b* values were immediately output on the display for further use. The degree of brightness was subtracted from the references (PP and PLA). Three samples were taken to obtain average values for each biocomposite material.

##### Tensile Strength and Young’s Modulus

The static mechanical properties of manufactured test specimens were measured in tensile strength and Young’s modulus according to DIN EN ISO 527-1 [39]. The tests were carried out on Zwick Roell Z020 universal testing machine. The testing speed for all measurements was 5 mm/min and it was 1 mm/min for the Young’s modulus. The reported values represent the averaged results of measurements performed on 10 samples for each type of composite material.

##### Impact Strength, IS

The impact strength (IS) was tested in accordance with DIN EN ISO 179-1 [40] on a Wick/Roell HIT 25P apparatus. All composites were tested at an ambient temperature of 23 °C and 50% relative humidity. The values presented comprise averaged results for 10 tests for each type of composite.

##### Heat Deflection Temperature, HDT

Heat deflection temperature (HDT) analysis was conducted according to ISO 75-1 [41]. The bending specimens were analyzed with 1.8 MPa bending force and a heating rate of 2 °K/min. HDT was measured when the specimen deflected by 0.34.

##### Differential Scanning Calorimeter, DSC

The melting temperatures of the composites and the neat matrix were investigated using 204 F1 Phoenix—Netszch Differential Scanning Calorimetry (DSC) attached with a cooling system under a nitrogen atmosphere. DSC analyses were conducted from 40 to 250 °C, with a heating rate of 10 °C/min (DIN EN ISO 11357-1) [42]. The specimens were sealed in aluminum pans by pressing and the prepared samples were placed in the furnace of DSC with an empty reference pan. The heat flow rate as function of temperature was recorded automatically. The melting temperature was identified as the peak point of the DSC curves.

##### Melt Flow Rate (MFR) and Melt Volume Rate (MVR)

MFR and MVR tests are crucial for assessing the processability, performance, and consistency of biocomposites made from Polypropylene (PP), polylactic acid (PLA), and natural fibers. The tests were performed on Aflow, ZwickRoell extrusion plastometers in accordance with ISO 1133-1:2011 [43] for PP-based biocomposites and ISO 1133-2:2011 for PLA-based biocomposites. The results of MRF and MVR were recoded as g/10 min and cm^3^/10 min, respectively.

##### Scanning Electron Microscopy—SEM

SEM micrographs of the fractured specimens were taken in order to evaluate the quality of the fiber–matrix interface. The sample fracture surfaces for this investigation were gold coated using a sputter coater (S150B, Edwards) to prepare the specimens. A scanning electron microscope EVO 60 of ZEISS, with an emission field gun with an acceleration voltage of 7 kV, was used.

## 3. Results and Discussion

### 3.1. Chemical Composition and Mechanical Properties of the Fibers

From the results presented in Table 1, it can be seen that kenaf has a high cellulose content (60%), moderate lignin content (22%), and wide variation in tensile strength (300–930 MPa) and a high tensile modulus (53 GPa). Bagasse exhibited lower cellulose content (40–50%), moderate lignin content (14.9%), a relatively low tensile strength range (20–50 MPa), and a low tensile modulus (2.7 GPa). As for the hemp, it had the highest cellulose content (70–74%) and lowest lignin content (3.5–5.7%), offering a high tensile strength range (550–900 MPa) and very high tensile modulus (70 GPa).

Hemp appears to offer the best mechanical properties, with a balance of high cellulose content and low lignin. Kenaf also has promising properties, while bagasse achieves a comparatively weaker performance. These properties are very important, as they have a direct effect on the mechanical properties of their composites. However, it should be noted that the best mechanical properties of the composite made from kenaf can be attributed to its high cellulose content, moderate lignin content, high tensile strength, high aspect ratio, and longer fiber length. These properties allow kenaf fibers to provide superior reinforcement and effective load transfer within the PP matrix. Bagasse, with its lower cellulose content and lower mechanical strength, followed by hemp, which has weaker fiber–matrix bonding due to its low lignin content, result in composites with lower mechanical properties.

### 3.2. Fiber Shaping

The results of the fiber shape analysis are shown in Table 2. It reveals that kenaf, bagasse, and soft wood are in the same range of 1 mm (mean value + standard deviation, SD). They also showed an equal length and thickness distribution (small size distribution), while hemp exhibited the smallest length and thickness.

Regarding the aspect ratio, the kenaf has the highest value and therefore also provides the largest available surface area. Thus, it is expected to be very reactive compared to others [44,45].

**Figure 3:**
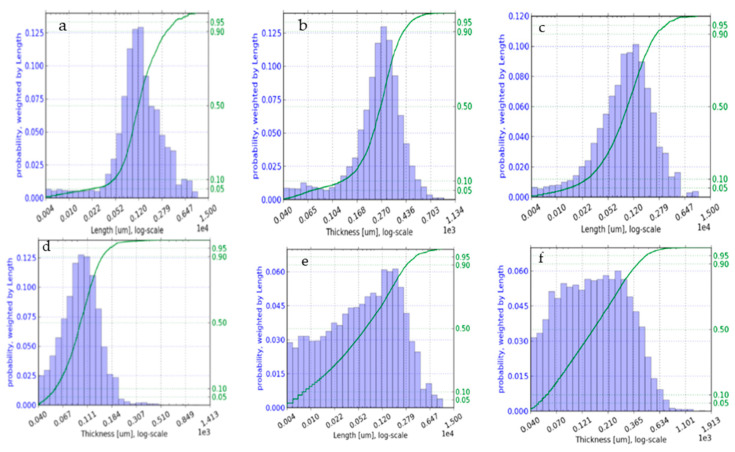
Images of length and thickness distribution of softwood (BK4090) (**a**,**b**), kenaf (**c**,**d**) and bagasse (**e**,**f**).

A clear peak can be observed for the kenaf and softwood in Figure 3. It indicates that they have similar fiber size distribution [46]. The bagasse has no clear peak; the distribution of length and thickness starts from 40 µm (minimum). Therefore, it has a big size distribution. Figure 1 compares the fiber length, thickness and aspect ratio for the four types of fibers used. The softwood had the longest and thickest fibers, followed by kenaf with regard to the fibers’ length and bagasse with regard to the fibers’ thickness. The aspect ratio (length-to-thickness ratio) is a critical factor that influences the mechanical and physical properties of fibers in bio-based composites. Kenaf has the highest aspect ratio, which is typically associated with improved mechanical properties, such as tensile strength, since longer fibers distribute stress more effectively in composites. Therefore, it is expected to exhibit the best mechanical performance. The aspect ratios of bagasse and softwood are comparable (Table 2), suggesting they may have similar mechanical properties. Hemp, with the lowest aspect ratio, was not expected to perform as well mechanically.

### 3.3. Density of the Composites

**Figure 4:**
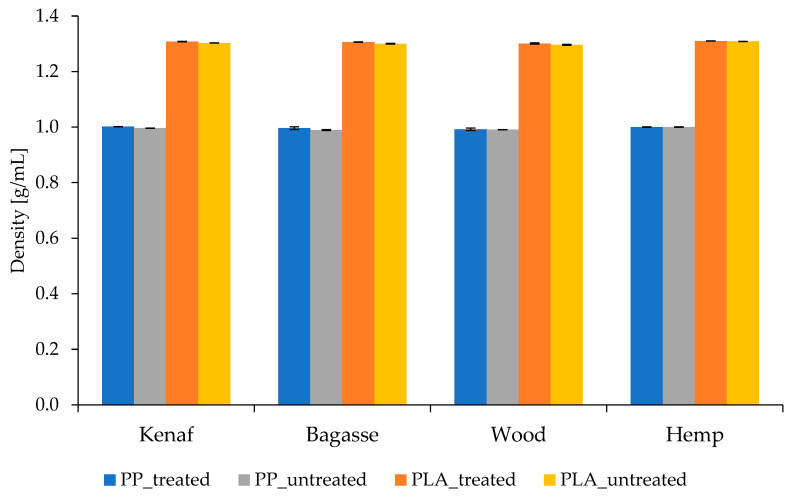
Comparison of density of composites from untreated and treated fibers with PP and PLA.

All manufactured biocomposites reinforced with fibers have density values in nearly the same range of 0.996–1.001 g/cm^3^ (see Figure 4) for the composites produced using PP. As for those produced by PLA, they exhibited densities ranging from 1.3 to 1.29 g/cm^3^, as shown in Figure 2. The density of polypropylene reinforced with glass fibers (PP–GF) typically falls within the range of 1.03 to 1.22 g/cm³ when considering standard fiber concentrations of 20 to 40 weight percent (wt%). One can observe that these densities are in the range of obtained densities for 20% of PP and glass fiber composites [47].

Low density of polymer composites filled with natural fibers was observed due to the specific hollow structure of the fibers, which is totally different from the bulky structure of glass fibers [48,49]. It has been observed that the fiber treatment did not affect the densities of the biocomposites. This could be related to the fact that as the treatment was mild, it affected the fibers’ surface, removing and modifying certain surface components like lignin, hemicellulose, or pectin, without significantly altering the overall mass or volume of the fibers.

Furthermore, the lower density of PP biocomposites compared to PLA is mainly due to the lower intrinsic density and simpler molecular structure of PP, which may not fully compensate for the added weight of the fibers, whereas the inherently denser PLA matrix contributes more to the overall weight of the composite. This density difference directly affects the mechanical and physical properties of the biocomposites, influencing factors like stiffness, thermal properties, and strength-to-weight ratio.

### 3.4. Brightness Measurement

**Figure 5:**
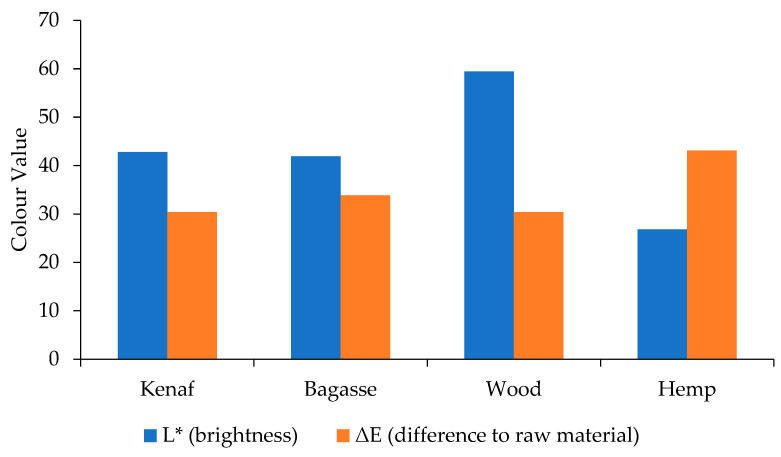
Color measurement results for the PP-treated fiber-reinforced composites.

**Figure 6:**
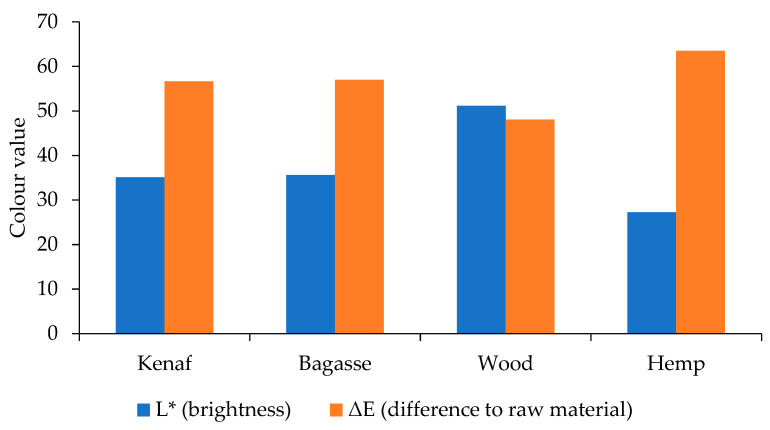
Color measurement results for the PLA-treated fiber-reinforced composites.

The results in Figure 5 and Figure 6 present the brightness and ∆E for both PP and PLA composites with neat PP and PLA as references. The results indicate that the wood + PP composites obtained the highest brightness (L*), while the hemp was the darkest one. The PLA composites followed the same trend as the PP composites.

The NaOH treatment of the wood removed a high amount of lignin and as a result, the produced composites obtained higher L* values than the untreated composites. As the color of natural fibers reverts to the lignin [50], the wood fibers and the natural fibers have different lignin structures and content [51,52,53]. Hemp has a relatively high lignin content compared to fibers like kenaf and bagasse, and lignin tends to darken under heat. This makes hemp-based composites appear darker. Even though the untreated fibers may not be dark, lignin degradation in the presence of heat can make hemp composites appear noticeably darker than those with kenaf and bagasse. Additionally, the thermal decomposition under processing condition contributes to color changes due to chromophores [54] formed from conjugated lignin structures. These factors collectively contribute to the darkening of biocomposites, with hemp composites often appearing the darkest among the three due to the hemp’s lignin content and thermal sensitivity.

### 3.5. Mechanical Properties

#### 3.5.1. Tensile Strength and Young’s Modulus

**Figure 7:**
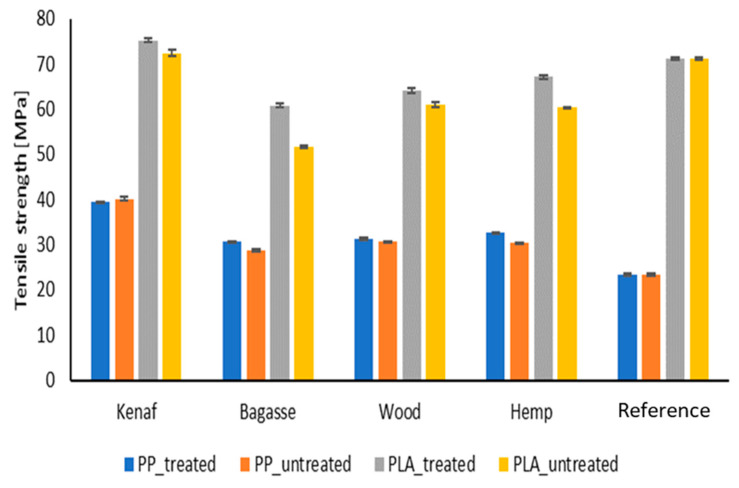
Tensile strength of treated and untreated PP and PLA fiber-reinforced composites.

**Figure 8:**
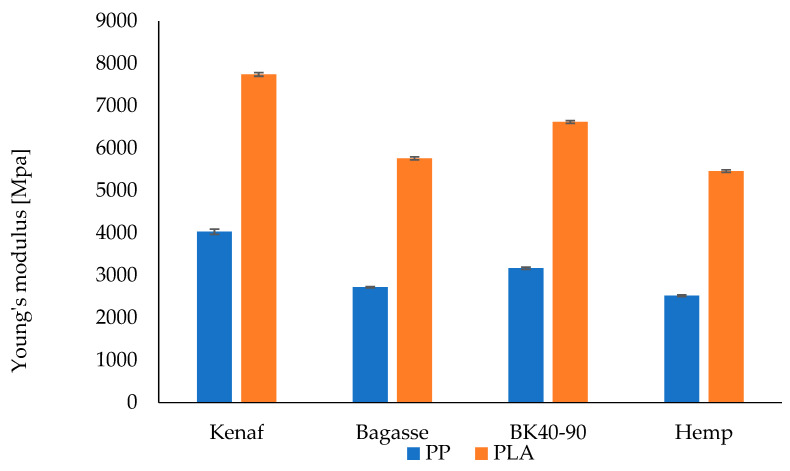
Young modulus of PP and PLA fiber-reinforced composites.

The experimental results of tensile strength and Young’s modulus for the PP and the PLA-based composites are shown in Figure 7 and Figure 8, respectively. The results show that incorporation of natural fiber mats has improved the tensile properties of neat PP for each type of composite. From the results, it can be observed that kenaf fiber-reinforced composites showed higher tensile strength (40.1 MPa) for both types of matrices as compared to other type of fiber reinforcement. PP/softwood, PP bagasse and PP/hemp composites showed an increased tensile strength of 30.1, 28.7 and 28.2 MPa, respectively. It is worth noting that a significant increase in mechanical properties is observed when comparing the biocomposites to pure PP (23.4 MPa) and PLA (71.2 MPa). However, there are no significant differences among the treated and untreated fiber PP biocomposites, which could be attributed to the mild fiber treatment which may not have substantially improved the fiber surface. The relatively low fiber content (25%) in this study could also influence the results. The observed improvement is likely due to the combination of the mild treatment and the intrinsic mechanical properties of the fibers themselves.

**Figure 9:**
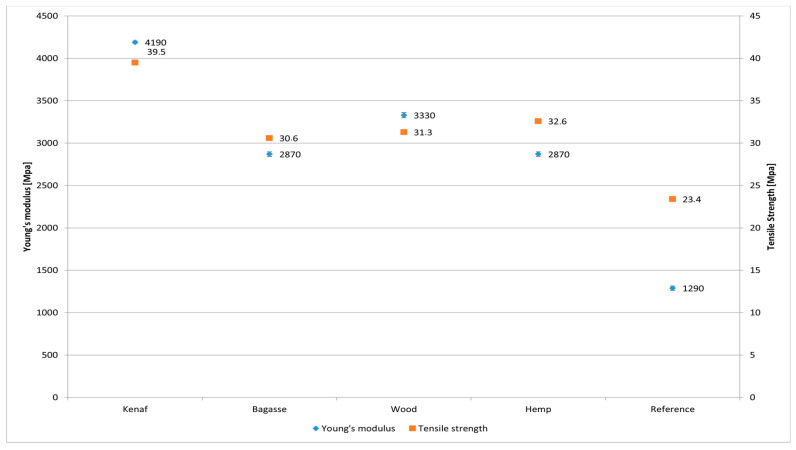
Comparison of tensile strength and Young’s modulus of PP-treated fiber-reinforced composites.

**Figure 10:**
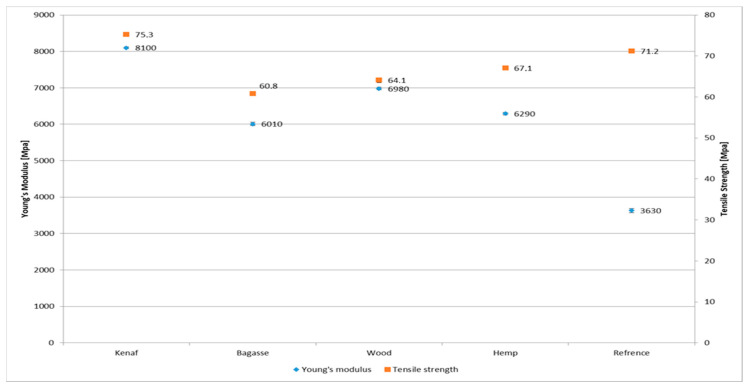
Comparison of tensile strength and Young’s modulus of PLA-treated fiber-reinforced composites.

Pure PP and pure PLA showed a Young’s modulus of 1290 and 3630 MPa, respectively. A slight increase in the Young’s modulus of all types of PLA-based composites can be observed in Figure 9 and Figure 10, whereas PP-based composites show a relatively higher increase. Although the PLA is more polar than PP, the addition of coupling agents (maleic anhydride-grafted PP) and influence of alkali treatment led to significant improvements in the fiber–matrix adhesion in the case of PP. It also resulted in better load transfer and a notable increase in tensile strength. The smaller increase in tensile strength when adding fibers to PLA compared to PP is due to the intrinsic mechanical properties of PLA, such as its brittleness and lower ductility, as well as differences in fiber–matrix adhesion and stress transfer efficiency. Hence, the alkali treatment might work effectively with PP compared to PLA [55,56]. Another aspect that should be taken into consideration when discussing the variation among the fibers is their chemical composition of cellulose and lignin content; however, this appears to also be connected to other factors such as the aspect ratio and the fibers length and thickness distribution. Although kenaf has lower cellulose content compared to hemp, it exhibited high mechanical performance that exceeded that of hemp fibers [57].

#### 3.5.2. Impact Strength (IS)

**Figure 11:**
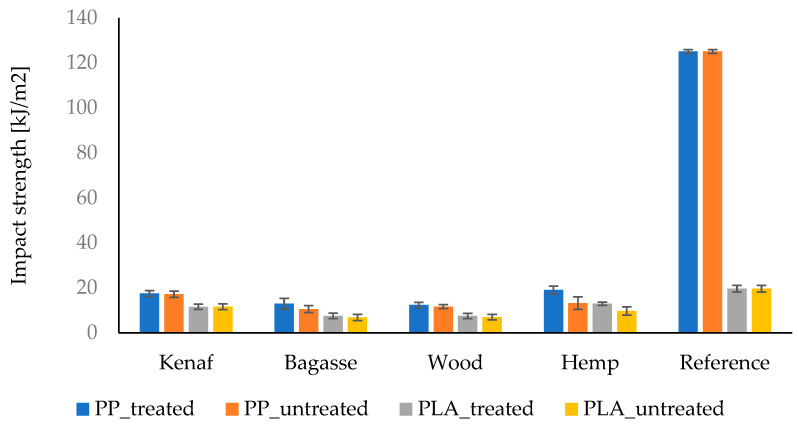
Comparison of the IS of treated and untreated fiber-reinforced PP and PLA composites.

The alkaline treatment of natural fibers could improve their composites’ mechanical properties depending on certain factors, such as the concentration of the NaOH and the soaking time [58] and the intrinsic properties of the fibers as well as the polymer metrics. The impact strength of the composites with treated and untreated fibers (Figure 11) showed close results in the most commonly produced composites, except the results obtained with the hemp fiber as filler. The hemp composites showed a clear improvement between the alkaline-treated composites and the untreated fibers, achieving the highest impact strength value. This result could be attributed to the fact that the hemp fibers’ thickness compared to the length distribution is uniform (see Table 2) compared to other fibers, which means hemp composites contain large quantities of thick fibers compared to the other types of composites [59,60]. The thicker fibers led to a good distribution which resulted in improved impact strength compared with the other fibers [61]. Furthermore, the alkaline treatment improved the bonding and surface roughness of hemp fibers as shown in Figure 11, allowing them to absorb more energy upon impact, even with a lower aspect ratio compared to the kenaf. Moreover, hemp fibers have higher toughness compared to other fibers, such as kenaf and wood. Toughness is a key factor in impact strength. The alkaline treatment tends to improve the toughness of hemp fibers more effectively because it optimizes the cellulose structure without significantly degrading the fiber [20,62,63,64,65].

### 3.6. Heat Deflection Temperature

**Figure 12:**
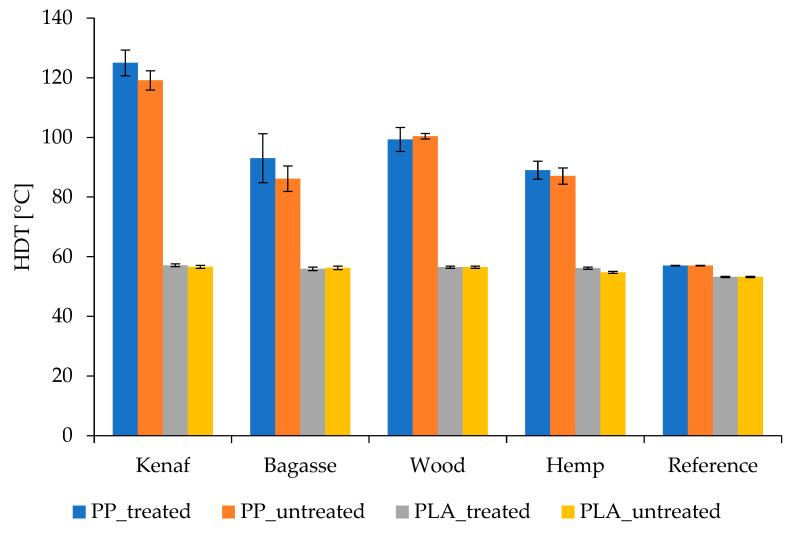
HDT of treated and untreated PP and PLA fiber-reinforced composites.

The interaction between the composite components affects the HDT of natural fiber plastic biocomposites. The addition of natural fiber to the thermoplastic polymers improves their HDT performance [66,67], as shown in Figure 12. It can generally be noticed that the PLA natural fiber composites have lower HDT values than the PP composites, and this is considered one of PLA’s weaknesses that affects its application specifically in a high-temperature environment [66,68]. On the other hand, the fibers treated with NaOH recorded slightly higher HDT values than untreated fibers in the PP composites. Meanwhile, in the PLA composites, there is no clear difference between the treated and untreated composites. Among all the prepared composites made with PP and treated kenaf fibers had the highest recorded HDT value, which was above 120 °C. This difference may be attributed to factors such as the inherent stability of treated kenaf (due to its high stiffness and rigidity) fibers, which contributes to improved thermal resistance, raising the HDT of the composite. Other factors could include alkali treatment of kenaf fibers typically removing surface impurities like lignin, wax, and oils, which improves the fiber–matrix adhesion by creating a rougher fiber surface. This stronger bonding allows a more efficient stress transfer between the fibers and the PP matrix under heat, maintaining structural integrity and resulting in higher HDT [55]. Furthermore, PP has higher compatibility with treated kenaf fibers compared to more hydrophilic fibers like hemp, which may have higher moisture absorption and, consequently, lower thermal stability [56]. The hydrophobic nature of the PP matrix complements the treated kenaf fibers, providing a better overall thermal performance [55].

### 3.7. Differential Scanning Calorimeter (DSC)

**Figure 13:**
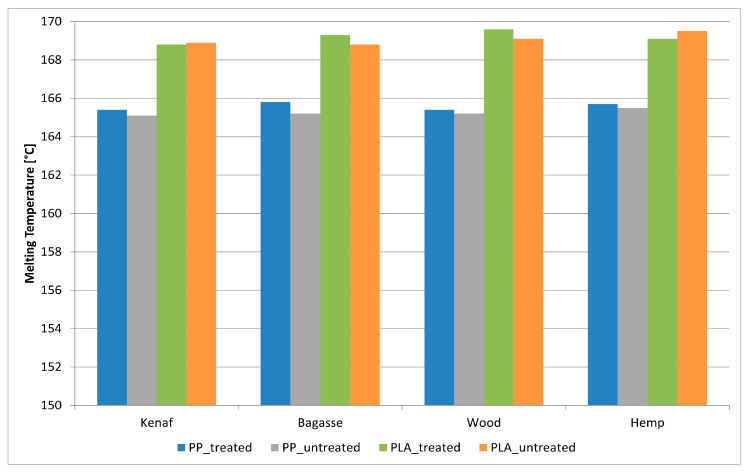
DSC of treated and untreated PP and PLA fiber-reinforced composites.

**Figure 14:**
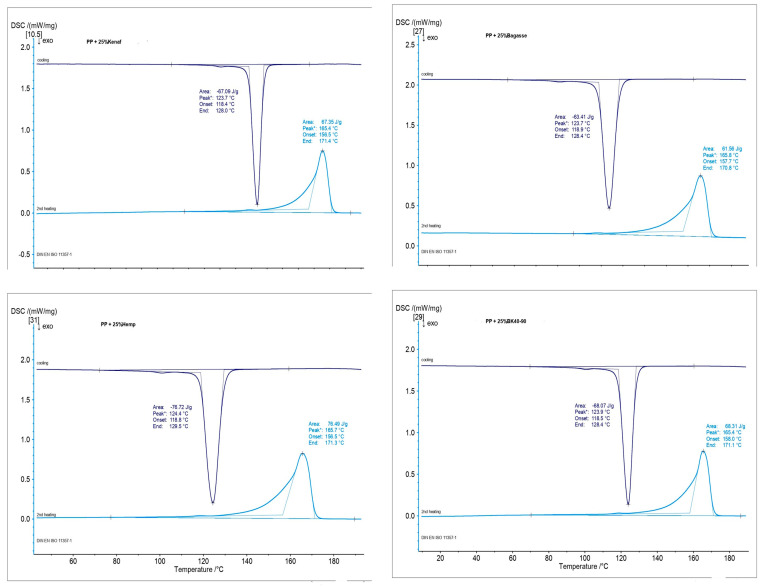
DSC of biocomposites made from PP and the treated fibers.

**Figure 15:**
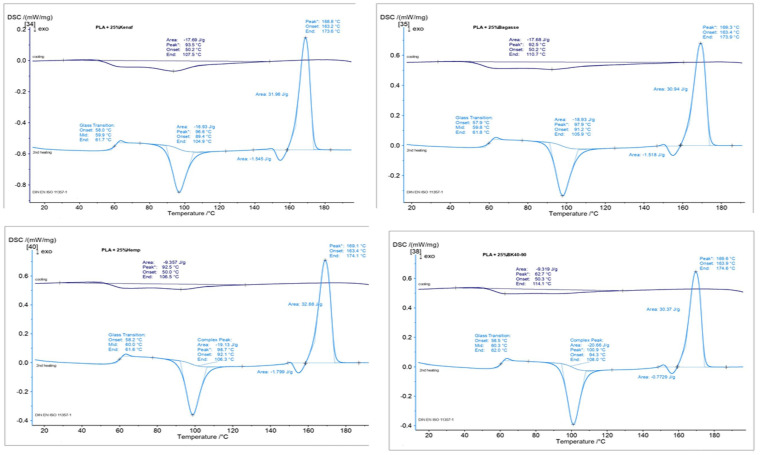
DSC of biocomposites made from PLA and the treated fibers.

The data presented in Figure 13, Figure 14 and Figure 15 illustrate the Differential Scanning Calorimetry (DSC) results for PP and PLA composites reinforced with treated and untreated natural fibers, including kenaf, bagasse, softwood, and hemp. The figure also highlights the impact of NaOH treatment on these natural fibers. A noticeable trend across all fibers is the slight increase in melting temperature after soft NaOH treatment, which suggests enhanced fiber–matrix interactions. This improvement is likely due to the treatment strengthening the bond at the fiber–polymer interface, thus stabilizing the polymer during melting. Among the fibers studied, bagasse shows the most significant improvement, followed by kenaf and hemp. These findings agree with those of [69,70], who reported that the alkaline treatment of the fibers leads to a fiber–matrix interface which also improves the thermal stability of the produced composites. It is worth noting that the thick, lignin-rich cell walls of bagasse make it particularly resilient to thermal degradation. Research has shown that lignin-rich, thick-walled fibers, such as those from bagasse, can improve the thermal stability of composites by acting as effective barriers to heat flow within the polymer matrix. Furthermore, the bagasse fibers contained parenchyma short fibers (spongy cells), which it used without depithing [71].

Another factor that can affect the melting temperature of composite materials is the release of fiber components into the matrix, which may occur at higher temperatures during extrusion. It has been observed that impurities can weaken the polymer lattice, making the polymer less stable and more susceptible to disruption. As a result, this leads to a lower melting point, a phenomenon known as melting point depression [72].

On the other hand, the DSC results of the different fiber-reinforced PLA composites in Figure 13 showcase the changes in melting temperature after NaOH treatment, revealing that the effectiveness of fiber alkaline treatment depends on the type of fiber. Bagasse and softwood have thicker fibers were that are flexible, which can limit movement within the polymer matrix and provide enhanced structural stability at elevated temperatures.

This means that with a greater increase in melting temperature, fibers’ morphology can play a significant role, in addition to the alkaline treatment, in terms of improving thermal stability in the produced composites. However, the minimal changes for kenaf and hemp indicate that their thinner fibers, together with the alkaline treatment, might not substantially alter their thermal properties. It appears that the fibers’ shape and the chemical composition and % of the added fibers have noticeable effects on the melting temperature of the composites.

### 3.8. Melt Flow Rate (MFR), and Melt Volume Rate (MVR)

**Figure 16:**
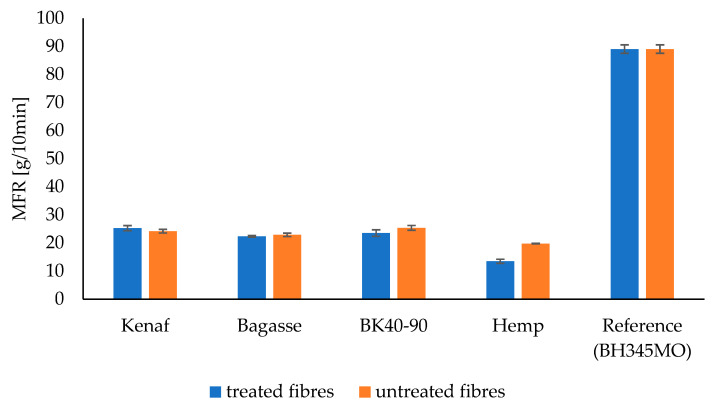
The melt flow rate (MFR) of the treated and untreated PP fiber-reinforced composites.

**Figure 17:**
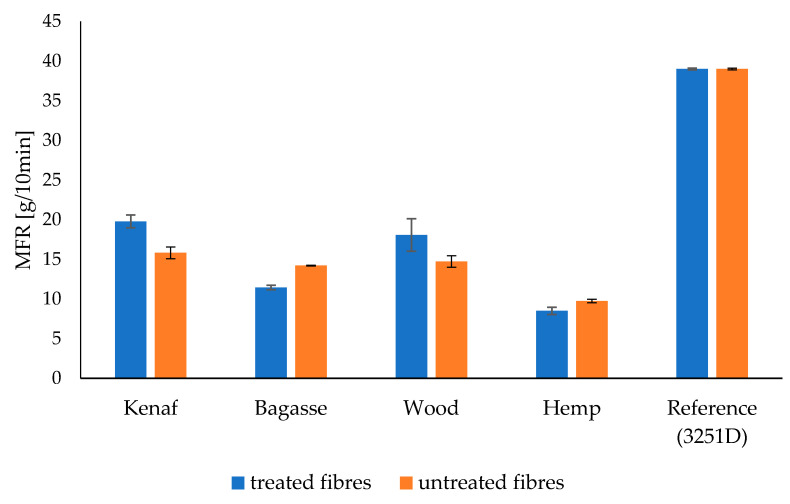
The Melt Volume Rate (MFR) of the treated and untreated PLA fiber-reinforced composites.

**Figure 18:**
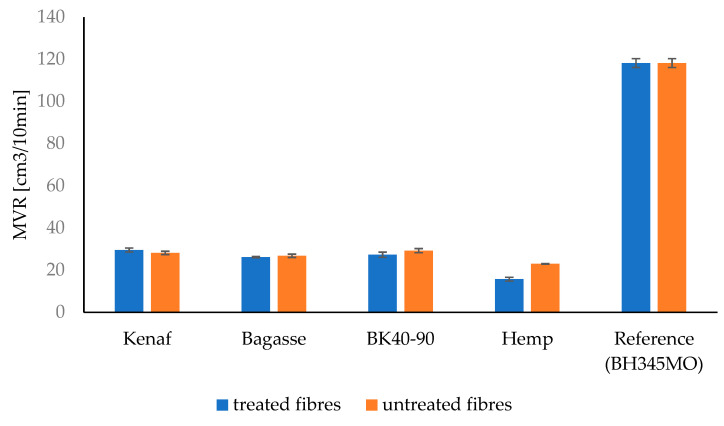
The Melt Volume Rate (MVR) of the treated and untreated PP fiber-reinforced composites.

**Figure 19:**
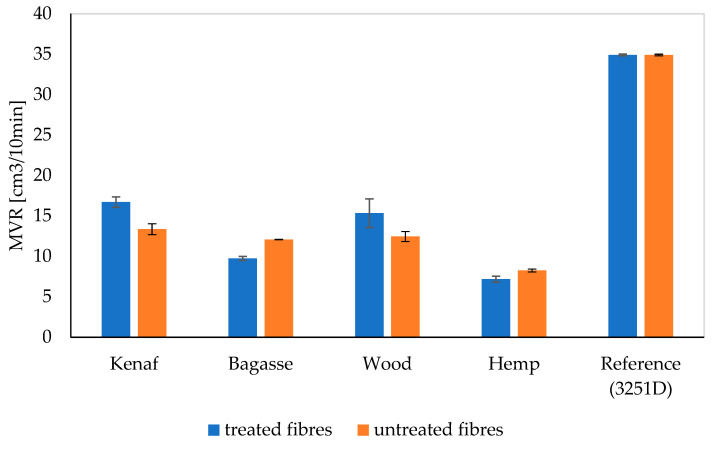
The Melt Volume Rate (MVR) of the treated and untreated PLA fiber-reinforced composites.

The MFR results for PP and PLA composites are presented in Table 3 and Table 4 and Figure 16 and Figure 17, while MVR is shown in Figure 18 and Figure 19, respectively. There is decrease in MFR values when fibers are added. This is understandable, as the presence of fibers in melts and their partial misalignment [73] significantly affects the dynamics of viscoelasticity of the melts [74], hindering the mobility of molecular chains due to polymer–particles interaction [75]. Furthermore, it can be seen that different fibers have different MFR values. Kenaf and softwood have comparable values due to the similarities in their fibers’ distribution and length.

The MFR and MVR graphs for PP composites reinforced with treated and untreated kenaf, bagasse, softwood, and hemp fibers show that the MFR of kenaf fibers treated with 5% NaOH is higher than that of the untreated kenaf in the PP matrix. However, for softwood, bagasse, and hemp, the MFR values for untreated fibers are slightly higher than those of the treated fibers.

The observed differences in the MFR and MVR of NaOH-treated versus untreated fibers in polypropylene (PP) composites can be attributed to the changes in fiber structure and interface characteristics after treatment. Alkaline treatment with NaOH is known to modify the surface of natural fibers by removing lignin, hemicellulose, and other impurities, which can improve fiber–matrix adhesion in PP composites. This enhanced adhesion often leads to a more effective stress transfer and reduced fiber sliding, resulting in a higher MFR compared to untreated fibers, particularly for fibers like kenaf. This suggests that kenaf fibers, after NaOH treatment, have better interfacial bonding with the PP matrix, which contributes to improved flow properties under the testing conditions.

However, for fibers like softwood, bagasse, and hemp, untreated fibers may sometimes show a slightly higher MFR. This can happen if the NaOH treatment reduces fiber flexibility or if certain structural components, such as lignin, which were partially retained in untreated fibers, improve processability without necessarily increasing matrix bonding as much as in the case of kenaf.

Such observations align with findings in polymer composites, where factors like fiber structure, treatment method, and fiber type play critical roles in MFR variation. This has been noted in studies on fiber-reinforced composites, including those incorporating lignocellulosic fibers like sisal and corncob in PP matrices, which showed treatment-induced changes in rheology and interface characteristics that could impact MFR depending on the fiber type and treatment intensity [76,77].

### 3.9. Morphological Analysis

**Figure 20:**
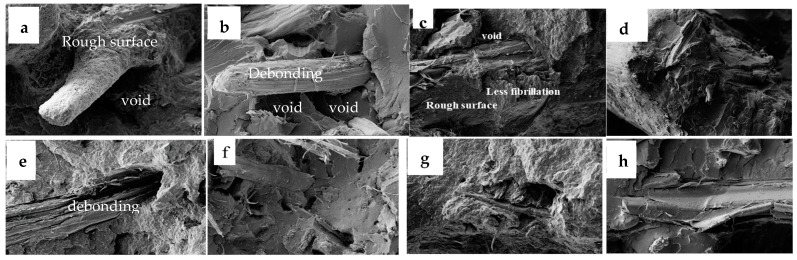
SEM micrographs (500×) of kenaf/PP biocomposites (**a**) kenaf/PLA biocomposite (**b**), bagasse/PP biocomposites (**c**), bagasse/PLA biocomposites (**d**), hemp/PP biocomposites (**e**), hemp/PLA biocomposites (**f**), softwood/PP biocomposite (**g**), and softwood/PLA biocomposite (**h**).

Morphological analysis of treated polypropylene (PP) and polylactic acid (PLA) biocomposites was conducted to evaluate fiber dispersion, fiber–matrix adhesion, and interfacial properties. SEM micrographs highlighted differences based on fiber type, polymer matrix, and treatment (Figure 20). The images of kenaf fibers embedded in PP (a) and PLA (b) reveal that NaOH treatment roughened the surface of the kenaf fibers by removing impurities, which improved mechanical interlocking with the PP matrix. This led to tighter bonding and fewer visible gaps, enhancing interfacial adhesion. In contrast, the image of PLA (b) composites showed weaker adhesion, as fibers appeared completely detached with residual PLA adhering to their surfaces. Voids were more prominent compared to the PP composite, indicating areas of debonding. The incompatibility, presence of large voids, and phase separation between the fibers and PLA matrix were attributed to the hydrophilic nature of kenaf fibers, which induced interfacial debonding with the hydrophobic PLA matrix. Similar results were observed by Moustafa et al. when using untreated kenaf fibers with polystyrene polymer [78]. The results indicate that treating kenaf fibers with NaOH does not significantly improve adhesion with the PLA matrix, likely due to the intrinsic incompatibility between the hydrophilic kenaf fibers and the hydrophobic PLA polymer. This suggests that additional strategies, such as using coupling agents or compatibilizers, might be necessary to promote better interfacial bonding with PLA [79].

For bagasse fibers, the SEM image of the PP composite (c) showed that the fibers exhibit a distinct texture compared to kenaf, presenting a more compact structure with less fibrillation. Bagasse fibers tend to maintain a more intact surface, unlike the roughened, fibrillated surface observed in treated kenaf fibers. These morphological differences influence their bonding with the polymer matrix, as reflected in the variation in mechanical properties. Consequently, kenaf demonstrated superior performance compared to bagasse. The micrographs of bagasse and PLA biocomposites (image d) revealed significant voids, gaps, and fiber pull-out compared to image (c), indicating poor interfacial adhesion and, consequently, subpar mechanical properties. Despite this, bagasse fibers have been shown to significantly enhance the stiffness of PLA even without the use of a coupling agent [80]. Similar studies suggested that adding a compatibilizer or hybrid materials such as nanosilica may improve adhesion [81].

The micrographs of hemp PP (e) and hemp PLA (f) composites revealed additional differences. Treated hemp PP composites exhibit good bonding between fibers and the matrix, with fewer gaps observed. The treatment resulted in smoother fiber surfaces and increased the aspect ratio of the fibers by reducing their thickness. This thinning, however, led to a decrease in the tensile strength of individual hemp fibers, ultimately resulting in lower mechanical properties for the hemp fiber composites [82]. In the case of PLA composites, significant fiber–matrix gaps indicate poor adhesion. Despite the NaOH treatment, rough fiber surfaces are still visible. Partially detached fibers and their imprints in the matrix are also noticeable, further suggesting weak interfacial bonding.

For softwood-based composites, the PP composite (g) showed pronounced surface roughness, with PP covering most of the fibers compared to image (h). However, some gaps and uncovered fibers remain visible. Regarding PLA composites, the extent of fiber pull-out is notably larger compared to PP composites, further underscoring the differences in interfacial adhesion between the two matrices. Moreover, the softwood-based PP and PLA biocomposites exhibited fibrous particles evenly dispersed within the PP and PLA matrices, with a minimal number of voids compared to the three other fibers. The fracture surfaces appeared uniform, indicating good adhesion. This may be attributed to the structure of wood particles, which lack lumens, unlike bast, stem, and leaf fibers [17]. It is worth noting that the micrograph of the softwood-PP biocomposite closely resembles that of the bagasse–PP biocomposite, indicating a similarity in their mechanical properties.

The differences between PP and PLA matrices were evident. PP, being a non-polar polymer, interacts weakly with natural fibers (which are polar). However, coupling agents such as maleic anhydride-grafted PP significantly improved fiber–matrix adhesion, leading to better load transfer and a notable increase in tensile strength. In contrast, PLA, a polar polymer, should theoretically exhibit better adhesion with polar natural fibers. However, PLA’s brittleness, rigidity, and crystalline structure limit its ability to distribute stress effectively, resulting in a smaller increase in tensile strength even with good bonding. Furthermore, the fracture behavior of the three different fiber types varied, influenced by their distinct mechanical properties and geometries, which affected stress distribution within the matrix.

## 4. Conclusions

The results demonstrate that fiber reinforcement significantly influenced the mechanical, physical, and thermal properties of PP and PLA biocomposites, with variations attributed to fiber geometry, aspect ratio, and alkaline treatment. The mechanical performance was highly dependent on fiber aspect ratio, with kenaf exhibiting the highest aspect ratio (12.27) and, consequently, the best tensile strength and Young’s modulus for untreated fibers (41.1 MPa and 4039 MPa, respectively) and treated fibers (39.5 MPa and 4190 MPa, respectively). Softwood and bagasse showed comparable performance, while hemp, with the lowest aspect ratio (5.66), had the weakest tensile strength for untreated fibers (30.4 MPa) and a slight improvement for treated fibers (32.6 MPa).

The density of the biocomposites ranged from 0.996 to 1.001 g/cm³, making them lightweight alternatives to synthetic fiber composites. Additionally, the chemical composition and alkaline treatment influenced color variations, with hemp displaying the darkest shade, providing esthetic versatility for eco-friendly designs.

In terms of thermal properties, kenaf-based PP composites exhibited the highest HDT for treated fibers (124.97 °C) compared to untreated fibers (119.13 °C). Bagasse and hemp had comparable melting temperatures of 165.8 °C and 165.7 °C, respectively, for treated fibers, which was attributed to their thick cell walls and high content of short fibers. PLA composites showed a slight improvement in HDT with fiber addition. Differential Scanning Calorimetry (DSC) revealed a slight decrease in melting temperature for PP composites and a slight increase for PLA composites.

Furthermore, fiber reinforcement reduced the melt flow rate (MFR) and melt volume rate (MVR) in both PP and PLA, as fibers hindered molecular chain mobility, affecting the viscoelastic behavior of the melts. While the alkaline treatment and high cellulose content played a role in fiber–matrix interactions, the findings suggest that fiber geometry had a more significant impact on mechanical, physical, and thermal properties.

Overall, this study highlights the potential of natural fibers, particularly kenaf and bagasse, in enhancing biodegradable composite performance, reinforcing their suitability for sustainable material applications. Future work will focus on fiber mapping and optimization of alkaline treatment parameters to further refine composite properties.

## Figures and Tables

**Table 1:** Chemical and mechanical composition of the fibers [31,34,35,36].

Fibers	Cellulose (%)	Lignin (%)	Tensile Strength (MPa)	Tensile Modulus (GPa)
Kenaf	60.00	22	300–930	53
Bagasse	40–50	14.90	20–50	2.7
Hemp	70–74	3.5–5.7	550–900	70

**Table 2:** Results of the fiber shape analysis for the untreated fibers.

Fibers	Length Mean Value [µm]	Thickness Mean Value [µm]	Aspect Ratio
Kenaf	1281.25 ± 17.1 *	104.42 ± 0.8 *	12.27
Bagasse	1175.29 ± 10.5 *	197.36 ± 1.5 *	5.96
Softwood	1638.13 ± 8.4 *	272.43 ± 2.3 *	6.01
Hemp	588.06 ± 3.2 *	103.95 ± 0.28 *	5.66

* Standard deviation.

**Table 3:** Results of MFR and MVR for the biocomposites made from the fibers and PP.

	Treated				Untreated				
	MFR		MVR		MFR		MVR		
Fibers	Mean	SD	Mean	SD	Mean	SD	Mean	SD	MC%
Kenaf	25.27	0.92	29.53	0.94	24.18	0.65	28.17	0.8	0.0194
Bagasse	22.36	0.29	26.18	0.3	22.87	0.61	26.8	0.78	0.0172
BK40-90	23.53	1.16	27.31	1.24	25.38	0.86	29.26	0.99	0.0172
Hemp	13.49	0.74	15.72	0.88	19.76	0.11	22.98	0.11	0.0255
PP	89	1.5	118.2	2.1	89	1.5	118.2	2.1	0.00

**Table 4:** Results of MFR and MVR for the biocomposites made from the fibers and PLA.

	Treated				Untreated				
	MFR		MVR		MFR		MVR		
Fibers	Mean	SD	Mean	SD	Mean	SD	Mean	SD	MC%
Kenaf	19.79	0.8	16.71	0.65	15.83	0.75	13.36	0.67	0.0194
Bagasse	11.45	0.28	9.74	0.25	14.22	0.05	12.09	0.03	0.0172
BK40-90	18.08	2.06	15.34	1.76	14.73	0.73	12.45	0.62	0.0172
Hemp	8.51	0.45	7.18	0.38	9.75	0.23	8.25	0.19	0.0255
PLA	39	0.1	34.9	0.1	39	0.1	34.9	0.1	0.00

## Data Availability

Data will be available upon request.
